# Supplementary material for: Reduced efficacy of a Src kinase inhibitor in crowded protein solution
Source: Nat Commun. 2021 Jul 2;12:4099. doi: 10.1038/s41467-021-24349-5 (PMC8253829; doi:10.1038/s41467-021-24349-5)
Supplement: Supplementary file 1 — Supplementary Information [file 41467_2021_24349_MOESM1_ESM.pdf]

Supporting Information for
Reduced Efficacy of a Src Kinase Inhibitor in Crowded Protein Solution

Kento Kasahara[a], Suyong Re[a], Grzegorz Nawrocki[b], Hiraku Oshima[a], Chiemi Mishima-Tsumagari[c], Yukako Miyata-Yabuki[c], Mutsuko Kukimoto-Niino[c], Isseki Yu[d], Mikako Shirouzu[c], Michael Feig[b,*], and Yuji Sugita[a,e,f,*]

[a]Laboratory for Biomolecular Function Simulation, RIKEN Center for Biosystems Dynamics Research, 6-7-1 Minatojima-minamimachi, Chuo-ku, Kobe, Hyogo 650-0047, Japan.

[b]Department of Biochemistry and Molecular Biology, Michigan State University, 630 Wilson Road, 218 Biochemistry Building, East Lansing, Michigan 48824-1319, United States.

[c]Laboratory for Protein Functional and Structural Biology, RIKEN Center for Biosystems Dynamics Research, 1-7-22 Suehiro-cho, Tsurumi, Yokohama, Kanagawa 230-0045, Japan.

[d]Department of Life Science and Informatics, Maebashi Institute of Technology, Kamisadori-machi, Maebashi, Gunma 371-0816, Japan.

[e]Computational Biophysics Research Team, RIKEN Center for Computational Science, 6-7-1 Minatojima-minamimachi, Chuo-ku, Kobe, Hyogo 650-0047, Japan.

[f]Theoretical Molecular Science Laboratory, RIKEN Cluster for Pioneering Research, Hirosawa 2-1, Wako, Saitama 351-0198, Japan.

[*]To whom correspondence may be addressed. Email:

Michael Feig (mfeiglab@gmail.com)

Yuji Sugita (sugita@riken.jp)

Supplementary Methods

The initial structure of c-Src kinase (Supplementary Figure 1) was prepared from an X-ray structure of unphosphorylated active-like c-Src kinase (PDB ID: 1Y57)[1]. Similar to the previous microsecond MD[2] and gREST/REUS simulations[3], only the kinase domain (residues 82-258) was used in the present simulations. For the dilute system, one c-Src kinase and 34 PP1 inhibitors were placed randomly, and then solvated by 150 mM NaCl aqueous solution. The crowded systems referred to as Src2BSA, Src4BSA, and Src8BSA also contain two, four, and eight BSA proteins (PDB ID: 4F5S)[4], respectively.

The protein volume fractions of Src2BSA, Src4BSA, and Src8BSA are 11%, 17%, and 30%. We used the AMBER ff99SB-ILDN[5,6] and TIP3P[7] parameters for the protein and water molecules. The PP1 parameters were obtained by GAFF with AM1- BCC[8]. In order to avoid PP1 aggregations, we adopt a repulsive potential between PP1 molecules[2]. The repulsive potential is defined as

$$U(r) = k(r - R)^2 \Theta(R - r),$$
$$k = 0.2 \text{ kcal mol}^{-1}, R = 20 \text{ Å},$$

where $\Theta(r)$ is the Heaviside step function. All bonds involving hydrogen atoms were constrained using the SHAKE algorithm[9] and water molecules were kept rigid using the SETTLE algorithm[10].

For each system, a set of seven different configurations was prepared. All the systems were initially equilibrated with NPT ensemble (Langevin thermostat and barostat, 310 K, 1 atm) for 1 ns using a development version of GENESIS package[11,12]. A friction parameter of 1 ps^{-1} was used for Langevin thermostat and barostat. A velocity version of Verlet integrator with a time step of 2 fs was used. After the equilibration, the NVT simulations were performed as the production simulations with GENESIS for six trajectories and for one trajectory with Anton2[13] implemented in Pittsburgh supercomputing center (PSC). For the GENESIS calculation, Bussi thermostat[14] was used for temperature control with a coupling time constant of 5 ps. An r-RESPA integrator with a time step of 2.5 fs was used. In the case of Anton2 calculations, the temperature was controlled with Nosé-Hoover thermostat (a time constant of 0.0412 ps). A multigrator algorithm with a time step of 2.5 fs was used. In all the analysis, the first 180 ns of each NVT trajectory was omitted as equilibration. The basic information on each system is shown in Supplementary Tables 1-3. To examine the effect of force field in use, we performed additional 1 μs MD simulations for dilute and Src8BSA systems using

CHARMM36m force fields and CHARMM compatible TIP3P water model[15] with all other settings the same as aforementioned simulations using GENESIS.

Analysis Details

The reference bound pose of PP1 to the canonical binding site of c-Src kinase

The reference structure of c-Src kinase-PP1 complex was obtained using two crystal structures: the autoinhibited hematopoietic cell kinase (Hck) complexed with PP1 (PDB ID: 1QCF)[16] and the unphosphorylated active-like c-Src kinase (PDB ID: 1Y57)[1], both of which were used in the previous computational work by Shan et al.[2] The PP1 of the former (1QCF) was aligned to the latter (1Y57) using backbone atoms of the binding pocket (residue 273, 274, 281, 293, 295, 323, 336, 338, 339, 340, 341, 344, 345, 393, 403 and 404). The coordinates of aligned PP1 and of the latter kinase structure were combined to get the reference structure of c-Src kinase-PP1 complex. (Supplementary Figures 1(a) and 1(b)). In the analysis of root mean square fluctuation (RMSF) and RMS displacement (RMSD), the residues 100-150, 168-200, and 225-259 in each of MD simulation snapshots were superimposed to the corresponding residues in the reference structure (Supplementary Figure 1(c)). The protein-PP1 distance, ξ, was defined as the distance between Center-of-Masses (CoMs) of PP1 and the backbone heavy atoms of 2 binding site residues (Ala35 and Leu135). The protein-PP1 distance in the reference structure is 3.59 Å (Supplementary Figure 1(d)).

Definition of reaction coordinates in the two-dimensional free energy landscapes

The PP1 position with respective to the canonical binding site of c-Src kinase was described using polar angles (θ, ϕ), which are determined using five anchor atoms (L0, L1, P1, P2, and P3)[3] as followed.

- L0: A non-hydrogen PP1 atom closest to the CoM of PP1.
- L1: CoM of L0 and non-hydrogen atoms of PP1 bonded to L0.
- P1: CoM of backbone heavy atoms of Tyr 82 (the residue closest to the CoM of the kinase).
- P2: CoM of backbone heavy atoms of Glu81 (which satisfies $30° \leq \mathrm{L1P1P2} \leq 150°$).

- P3: CoM of backbone heavy atoms of Val79 (which satisfies the $30° \leq \angle\mathrm{P1P2P3} \leq 150°$).

These anchor atoms were defined using the aforementioned reference structure of c-Src kinase-PP1 complex, and used throughout the analysis. The polar angles are defined as $\theta = \angle\mathrm{P2P1L1}$ and $\phi = \angle\mathrm{P1P2P3}$. Using these angles, together with the protein-PP1 distance ξ, two-dimensional free-energy landscapes were built from the previous our gREST/REUS simulations[3] with restraints imposed on c-Src kinase (restraint) and without any restraints (free) as shown in Supplementary Figure 14.

<u>The classification of the active/inactive states of c-Src kinase</u>

Two pseudo-torsion angles in DFG (Asp146-Phe147-Gly148) motif were used to classify the active and inactive kinase conformations, which was used in the previous work by Möbitz[17].

- $\zeta(\mathrm{DFG\!-\!Phe} \ldots \mathrm{DFG\!-\!Gly})$: The torsion angle composed of Cα atoms of Asp146, Phe147, Gly148, and Leu149.
- $\zeta(\mathrm{DFG} - 1 \ldots \mathrm{DFG\!-\!Asp})$: The torsion angle composed of Cα atoms of Val144, Ala145, Asp146, and Phe147.

The arguments in ζ indicate the central pairs of residues in the torsional angles. DFG–1 designates the residue next to Asp146 toward N-terminal, which is Ala145 in the present system. The distribution of the conformations along the two torsion angles (DFG-plot) is shown in Supplementary Figure 4.

<u>Definition of surface regions on proteins and the water phase</u>

In the spatial partitioning of PP1, the surface region on proteins and the water phase are defined according to the distance between PP1 and proteins (Supplementary Figure 8). The surface region on protein α (α = c-Src kinase, BSA) is defined if the minimum distance between PP1 (L0 atom) and heavy atoms of the protein α is shorter than 5 Å. The region outside the surface region is defined as the water phase.

<u>Spatial distribution functions (SDFs)</u>

The SDFs of PP1 near either c-Src kinase (Figure 1(a), Supplementary Figure 5) or BSA (Supplementary Figure 7) were computed using Volmap plugin implemented in VMD[18].

To calculate the functions, the configuration of either c-Src kinase or BSA in each snapshot form the simulation trajectory was aligned to the crystal structure. After the alignment, the occupancy of PP1 (L0 atom) in each of the rectangular grids (grid spacing is 0.5 Å in each direction) was calculated. The SDF of BSAs (Cα atoms) was computed in the same way (Figure 1(b), Supplementary Figure 6).

Spatial decomposition of mean square displacements (MSDs)

The heterogeneity of the PP1 diffusion depending on the environments (c-Src kinase surface, BSA surface, and the water phase) was examined by calculating the PP1 MSD for each region (Supplementary Figure 11). We first divided the trajectories into the bunch of short trajectories based on the locations of each PP1, and then calculated the time series of PP1 displacements for each trajectory. Finally, the MSDs were obtained by averaging the displacements over the short trajectories for each PP1 location. Similarly, the MSD in the water phase was also calculated (Supplementary Figure 21).

Residence time correlation functions of PP1

The residence time constants of PP1 on the surface of either c-Src kinase or BSA were obtained by calculating the time correlation functions for surface region α, $f_\alpha(t)$, as proposed by Impey *et al*[19]. The definition of $f_\alpha(t)$ is

$$f_\alpha(t) = \frac{\langle P_\alpha(t_{\mathrm{init}}, t_{\mathrm{final}}; t^*)\rangle_{t_{\mathrm{init}}}}{\langle P_\alpha(t_{\mathrm{init}}, t_{\mathrm{tinit}}; t^*)\rangle_{t_{\mathrm{init}}}},$$

where $t = t_{\mathrm{final}} - t_{\mathrm{init}}$. $P_\alpha(t_{\mathrm{init}}, t_{\mathrm{final}}; t^*)$ is the probability of finding PP1 which stays in the surface region α from t_{init} to t_{final}. We allowed temporary excursion of PP1 to the water phase for a time interval no longer than t^*. In the present study, the value of $t^* = 0.96$ ns was used for both the c-Src kinase and BSA cases. $\langle \cdots \rangle_{t_{\mathrm{init}}}$ indicates the ensemble average at the initial time t_{init}. To obtain the residence time constants, the $f_\alpha(t)$ was fitted to a triple exponential function with three time-constants (τ_1, τ_2, and τ_3) as well as two coefficients (A and B)

$$f_\alpha(t) \sim Ae^{-t/\tau_1} + Be^{-t/\tau_2} + (1 - A - B)e^{-t/\tau_3}.$$

The obtained values are listed in Supplementary Table 6. τ_1, τ_2, and τ_3 correspond to the fast, intermediate, and long time-scale diffusion of PP1, respectively.

Binding free energy estimation of Src-PP1 and BSA-PP1 complexes for different binding sites

The binding free energies of PP1 to the potential binding sites of Src-PP1 and BSA-PP1 were obtained through the molecular mechanics with generalized Born surface area (MM-GBSA) analysis using AmberTools18[20]. We first aligned each snapshot from the trajectories to the crystal structure of c-Src kinase or BSA. Then, we extracted the snapshots in which PP1 is in the protein surface region using aforementioned definition. Finally, the position of bound PP1 (L0 atom) was classified using *k*-means++ clustering implemented in Scikit-learn. The number of clusters for c-Src kinase and BSA were set to 25 and 30, respectively. The protein-PP1 complex was classified as bound state *i*, if the distance between the bound PP1 (L0 atom) and cluster center *i* is shorter than 4.5 Å. The MM-GBSA analysis was performed for each bound state *i*. For each state, ~1% snapshots is used for the analysis. The number of snapshots for each state is 15, 32, 14, 125, 85, 23, 68, 143, 53, 18, 9, 23, 57, 56, 68, 62, 14, 78, 33, 40, 15, 19, 12, 14, and 19 for c-Src-PP1 complex, and 4, 12, 94, 172, 157, 31, 33, 75, 5, 31, 181, 106, 65, 12, 43, 86, 37, 11, 100, 9, 12, 7, 19, 13, 15, 98, 6, 16, 31, and 11 for BSA-PP1 complex. The averaged value of each state is shown in Supplementary Figure 9. We used the AMBER ff99SB forcefield for this analysis.

The simulations starting from encounter states, **E** and $\mathbf{E_r}$

The 30 additional MD simulations in Src8BSA starting from starting from **E** (Supplementary Figure 12) and $\mathbf{E_r}$ (Supplementary Figure 13) states were performed. The 30 simulations were started by assigning the different initial velocities to all the atoms in the systems using GENESIS. We equilibrated the systems for 1 ns in NVT ensemble in which the harmonic restraint positional was applied to the PP1 (L0 atom) with the force constant of 10 kcal/mol/Å^2. Subsequently, additional 1 ns NVT simulations were performed without the restraints. Finally, the 20-ns NVT simulations were conducted for the analysis. We observed three binding events in the simulations started from $\mathbf{E_r}$ state, while no binding events was observed in the simulations from **E** state. The obtained binding trajectories projected onto the PMF (restraint) are shown in Supplementary Figure 17.

Contact analysis between c-Src kinase and BSA for TYR-in/out states

The difference in the c-Src kinase-BSA interactions between TYR-in and TYR-out conformations was analyzed by counting the number of contacts between two proteins in each conformation. We defined the TYR-in state as if Tyr82-Gly86 distance is shorter than 6.5 Å, and TYR-out otherwise. We consider that residue *i* in c-Src kinase is in contact with BSAs if the minimum distance between Cα atom of residue *i* in c-Src kinase and Cα atoms of BSAs is shorter than 10 Å. The number of contacts were then averaged over all the snapshots (Supplementary Figure 23).

Supplementary Table 1: Information on each MD simulation. The simulation ID (Sim. ID, D-: dilute, C-: crowded) with 'G' and 'A' indicates the trajectories obtained from GENESIS and Anton2, respectively. Asterisk in ID indicates the observations of PP1 binding to the canonical site in c-Src kinase in the simulations.

	Dilute				Src2BSA (11%)		
Sim. ID	Simulation	Binding	Time length / μs	Sim. ID	Simulation	Binding	Time length / μs
D-1G	GENESIS	No	2.1	C2-1G	GENESIS	No	1.0
D-2G	GENESIS	No	2.1	C2-2G	GENESIS	No	1.0
D-3G	GENESIS	No	2.1	C2-3G	GENESIS	No	1.0
D-4G*	GENESIS	Yes	2.1	C2-4G	GENESIS	No	1.0
D-5G*	GENESIS	Yes	2.1	C2-5G	GENESIS	No	1.0
D-6G*	GENESIS	Yes	2.1	C2-6G	GENESIS	No	1.0
D-7A*	Anton2	Yes	20.0	C2-7G	Anton2	No	10.0
	Src4BSA (17%)				Src8BSA (30%)		
Sim. ID	Simulation	Binding	Time length / μs	Sim. ID	Simulation	Binding	Time length / μs
C4-1G	GENESIS	No	1.0	C8-1G	GENESIS	No	2.1
C4-2G	GENESIS	No	1.0	C8-2G	GENESIS	No	2.1
C4-3G	GENESIS	No	1.0	C8-3G	GENESIS	No	2.1
C4-4G	GENESIS	No	1.0	C8-4G	GENESIS	No	2.1
C4-5G	GENESIS	No	1.0	C8-5G	GENESIS	No	2.1
C4-6G	GENESIS	No	1.0	C8-6G*	GENESIS	Yes	2.1
C4-7A	Anton2	No	10.0	C8-7A*	Anton2	Yes	20.0

Supplementary Table 2: Box length and numbers of atoms, BSA, and PP1 in each system.

	Box length / Å	# of atoms	# of BSA	# of PP1	volume fraction / %
Dilute	136.0	245876	0	34	0
Src2BSA	136.3	250465	2	32	10.8
Src4BSA	136.6	255044	4	31	16.9
Src8BSA	135.7	255373	8	25	20.6

Supplementary Table 3: Concentrations of c-Src kinase, BSA, and PP1 in each simulation system. The concentration in g/L is shown in parentheses.

	[c-Src] / mM (g/L)	[BSA] / mM (g/L)	[PP1] / mM (g/L)
Dilute	0.69 (22)	0	24 (6.6)
Src2BSA	0.74 (23)	1.5 (98)	24 (6.6)
Src4BSA	0.78 (25)	3.1 (207)	24 (6.6)
Src8BSA	1.00 (30)	7.6 (501)	24 (6.6)

Supplementary Table 4: The probability of finding PP1 in the bulk water region, on the surfaces of c-Src kinase or BSAs. The values in parentheses indicate the probability of finding PP1 on single BSA.

	Dilute	Src2BSA	Src4BSA	Src8BSA
c-Src kinase (%)	20.1 ± 0.9	16.5 ± 3.1	11.0 ± 3.1	8.3 ± 4.3
BSA (%)	0.0	55.3 ± 3.1 (27.7 ± 1.5)	80.2 ± 4.6 (20.0 ± 1.2)	89.9 ± 4.4 (11.2 ± 0.5)
Bulk Water (%)	79.9 ± 0.9	28.1 ± 3.2	8.8 ± 2.4	1.8 ± 0.4

Supplementary Table 5: The probability of finding PP1 per unit surface area on c-Src kinase or BSA (10^{-4} % /Å^2).

	Dilute	Src2BSA	Src4BSA	Src8BSA
c-Src kinase	14.7 ± 0.7	12.1 ± 1.4	8.1 ± 2.3	6.1 ± 3.2
BSA	0.0	10.4 ± 0.6	7.5 ± 0.4	4.2 ± 0.2

Supplementary Table 6: Residence time constants for PP1 on the surfaces of c-Src kinase (Src-PP1) and BSA (BSA-PP1) obtained from the curve fitting of the residence time correlation functions. The triple exponential function ($f(t) = Ae^{-t/\tau_1} + Be^{-t/\tau_2} + (1 - A - B)e^{-t/\tau_3}$) is used for the fitting.

	Src-PP1				
	τ_1 / ns	τ_2 / 10^1 ns	τ_3 / 10^2 ns	A	B
Dilute	1.47	1.30	0.73	0.56	0.33
Src2BSA	1.40	1.57	1.12	0.46	0.38
Src4BSA	1.46	1.13	0.98	0.43	0.36
Src8BSA	1.25	1.25	1.04	0.63	0.24
	BSA-PP1				
	τ_1 / ns	τ_2 / 10^1 ns	τ_3 / 10^2 ns	A	B
Src2BSA	1.57	1.38	1.63	0.59	0.33
Src4BSA	1.29	1.24	1.65	0.63	0.26
Src8BSA	1.16	1.26	1.64	0.65	0.25

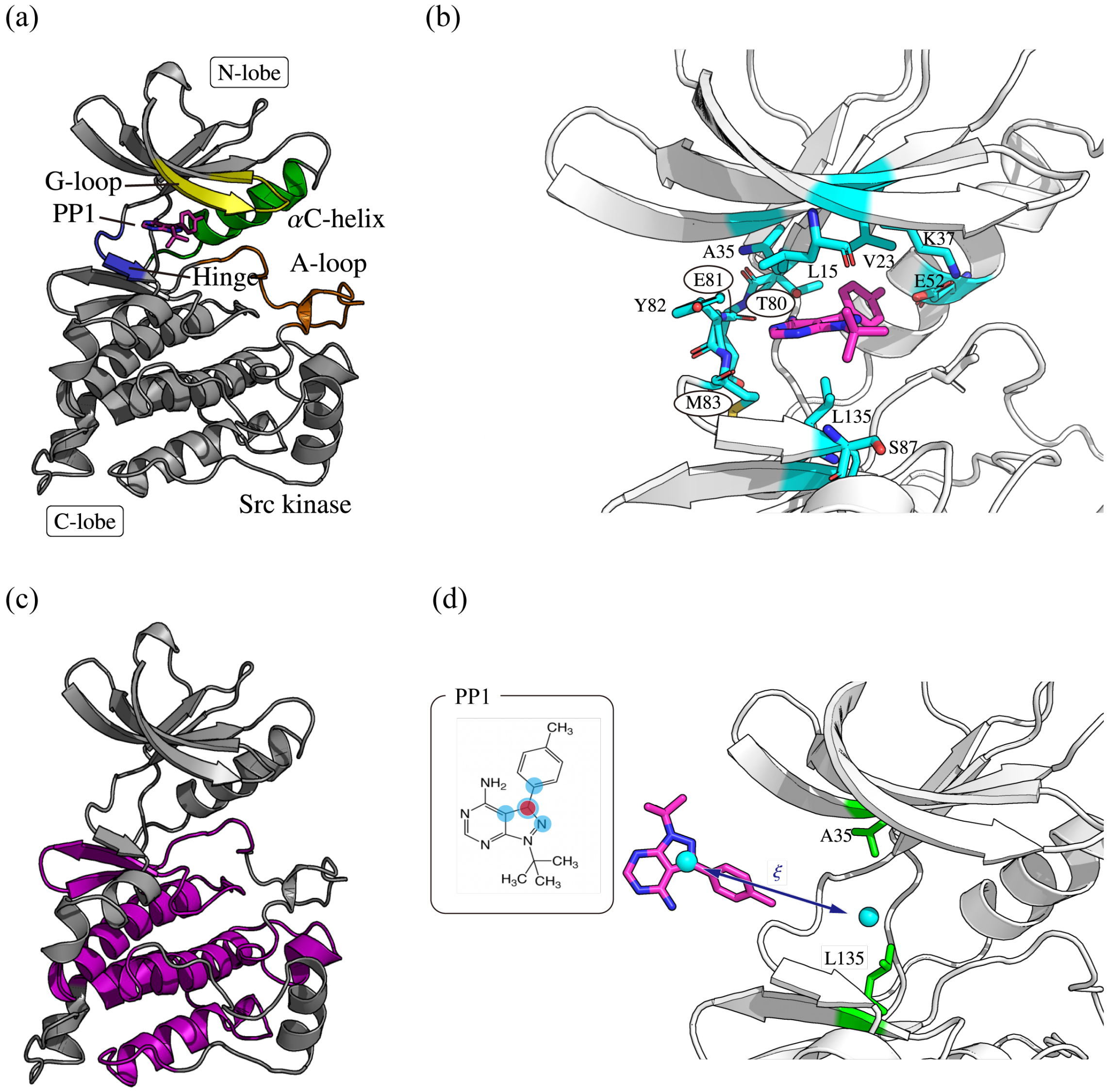

Supplementary Figure 1: (a) The crystal structure of the c-Src kinase-PP1 inhibitor complex. The structure was built using the X-ray structures of the c-Src kinase (PDB ID: 1Y57) and of the autoinhibited form of Hck complexed with PP1 (PDB ID: 1QCF). (b) A close up view of the canonical binding site (ATP binding site) with bound PP1. The circled residues (Glu81, Thr80, and Met83) form the hydrogen bonds to PP1 (c) The residues 100-150, 168-200, and 225-259 (colored in purple) in MD simulation snapshots used to superimpose to those in the crystal structure in the analysis of root mean square fluctuation (RMSF) and RMS displacement (RMSD). (d) Definition of the protein-PP1 distance, ξ.

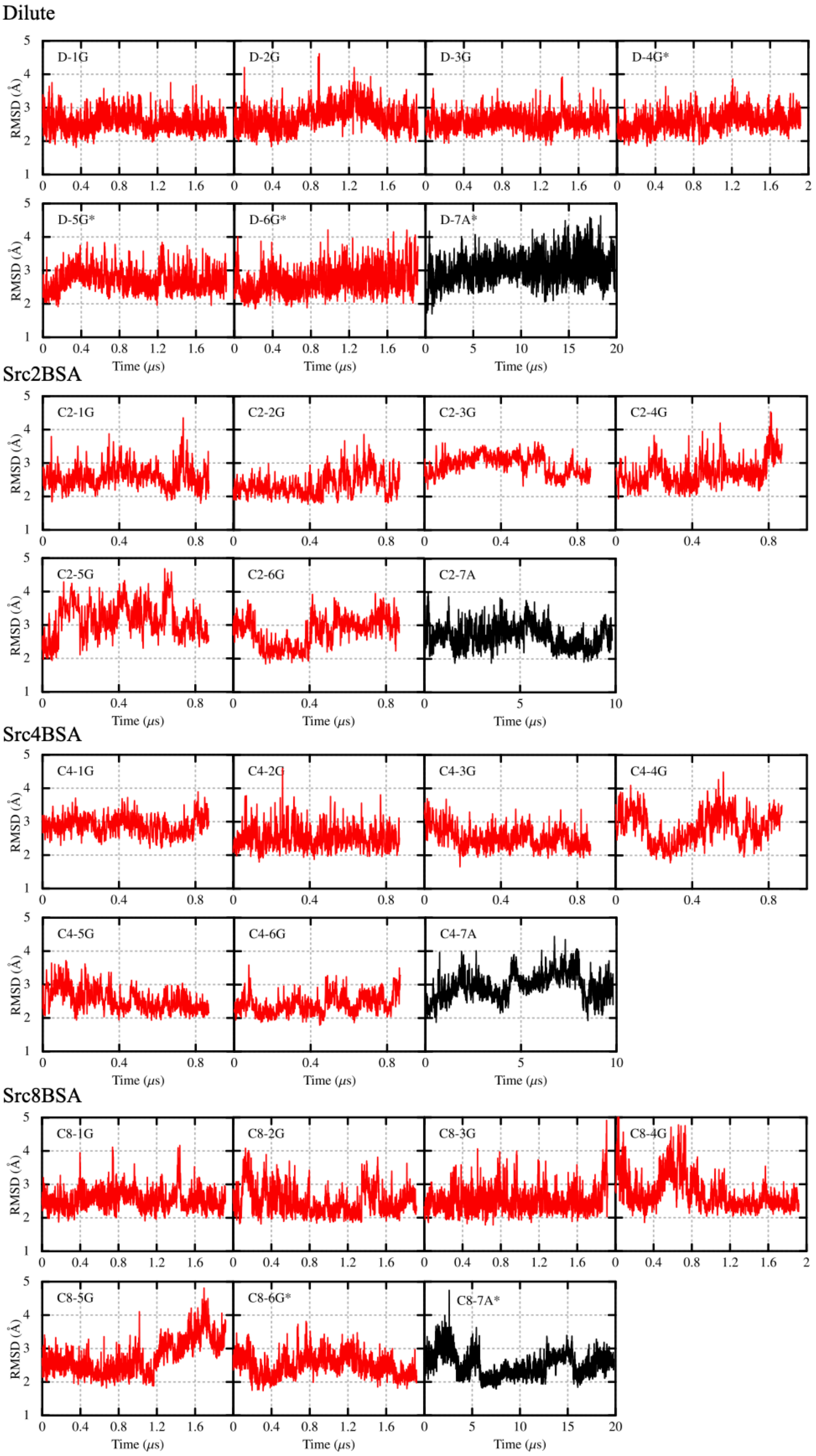

Supplementary Figure 2: RMSD of the Cα atoms in c-Src kinase from the crystal structure (PDB ID: 1Y57). The residues near the N-terminal, C-terminal, and in the A-loop are omitted in the analysis. The red lines were obtained using GENESIS, while the black ones using Anton2 in Pittsburg supercomputer center.

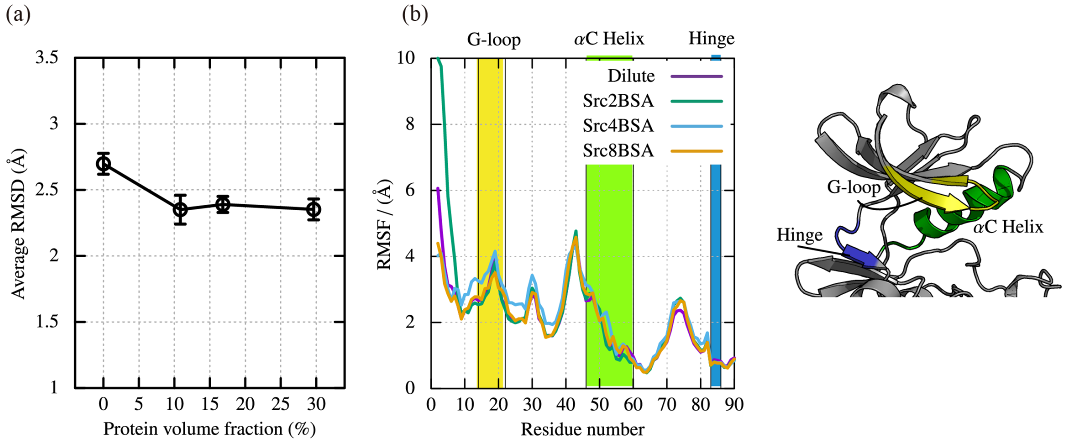

Supplementary Figure 3: (a) Time averages of RMSDs of the Cα atoms in c-Src kinase from the crystal structure (PDB ID: 1Y57). (b) RMSFs of c-Src kinase in the unbound state. The unbound state is defined if the protein-PP1 distance, ξ, is longer than 15 Å. G-loop, αC Helix, and Hinge regions are colored in yellow, green, and blue, respectively. Error bars of (a) represent the standard error of the mean from seven trajectories for each system.

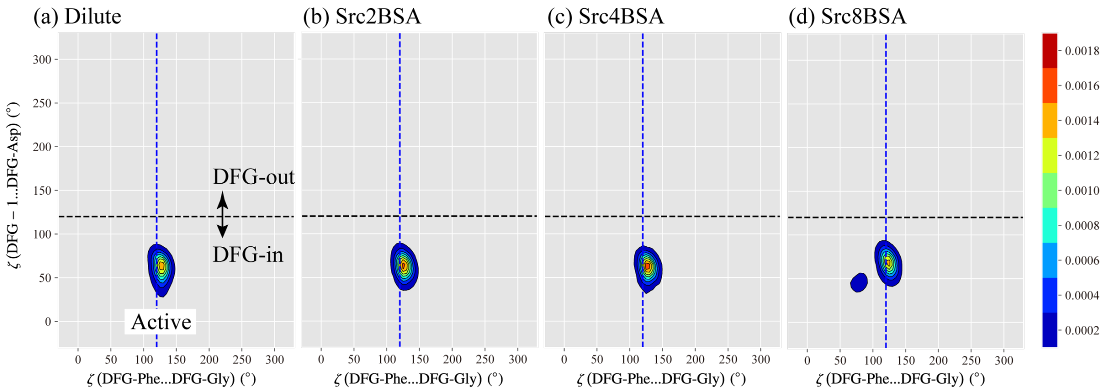

Supplementary Figure 4: The distribution of c-Src kinase conformations along the two pseudo-torsion angles related with DFG-motif, $\zeta(\text{DFG–Phe} \ldots \text{DFG–Gly})$ and $\zeta(\text{DFG} - 1 \ldots \text{DFG–Asp})$. Black-dotted line ($\zeta(\text{DFG} - 1 \ldots \text{DFG–Asp}) = 120°$), and blue-dotted line ($\zeta(\text{DFG–Phe} \ldots \text{DFG–Gly}) = 120°$) in the figures indicate respectively the boundary of DFG-in/DFG-out states, and αC-out state, as proposed by Möbitz[17].

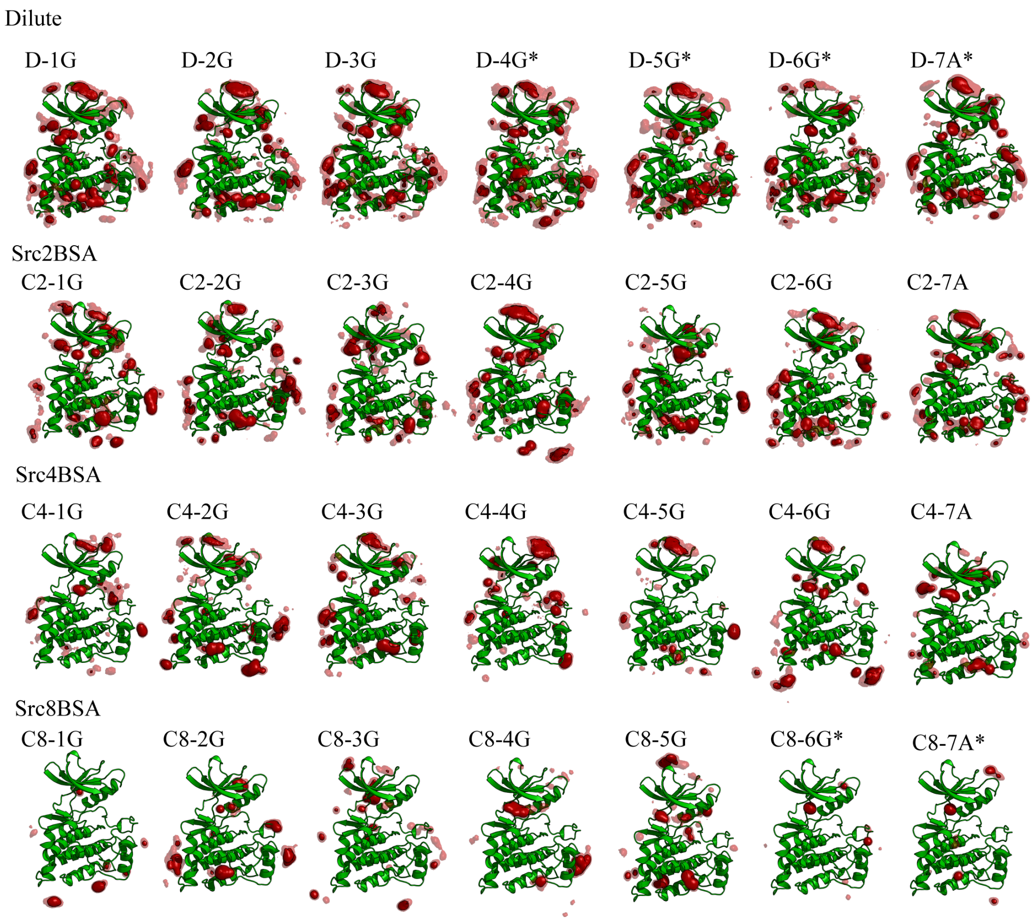

Supplementary Figure 5: Spatial distribution functions (SDF) of PP1 around c-Src kinase obtained in each MD trajectory. C2-7A, C4-7A, and C8-7A were obtained in Anton2 simulations.

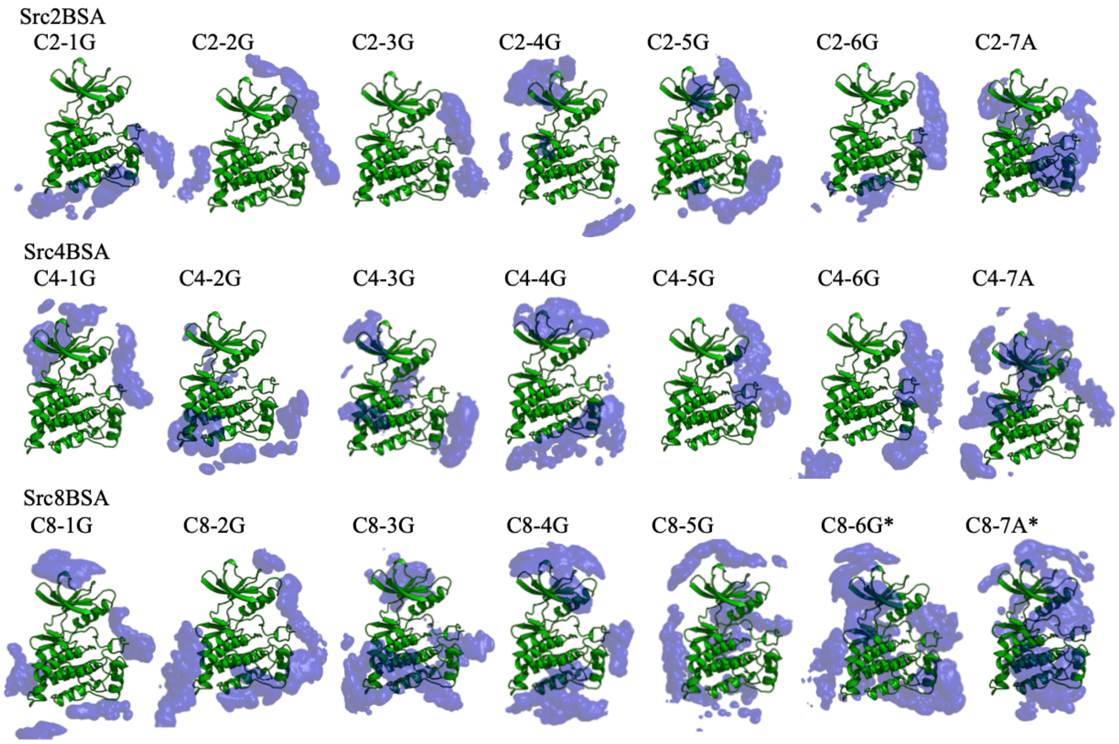

Supplementary Figure 6: Spatial distribution functions (SDF) of the Cα atoms in BSA around c-Src kinase obtained in each MD simulation with BSA crowders (Src2BSA, Src4BSA, and Src8BSA). C2-7A, C4-7A, and C8-7A were obtained in Anton2 simulations.

(a)

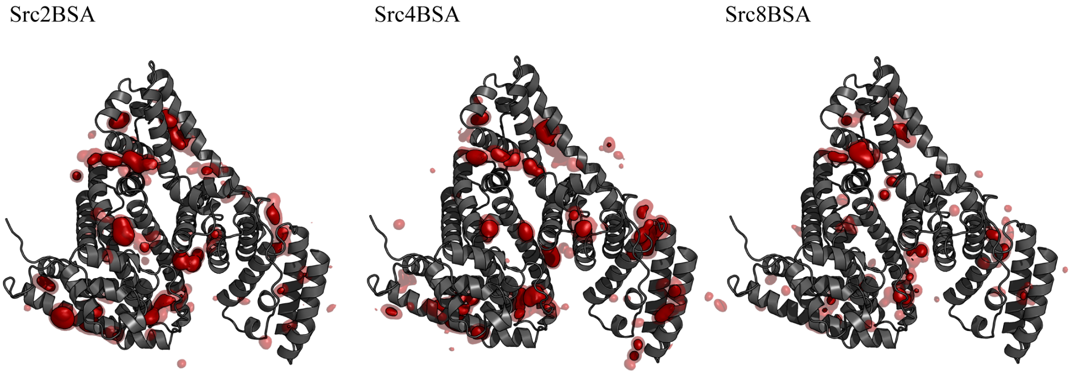

(b)

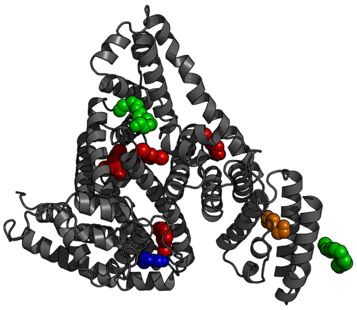

Supplementary Figure 7: (a) Spatial distribution functions (SDF) of PP1 around BSA obtained in the MD trajectories with BSA crowders (Src2BSA, Src4BSA, and Src8BSA). (b) The reported bounded poses of the other compounds in the crystal structure (PDB ID: 4JK4)[21]. The compounds, 2-hydroxy-3,5-diiodo-benzoic acid, di(hydroxyethyl)ether, penta-ethylene glycol, and tri-ethylene glycol are colored in red, blue, green, and orange, respectively.

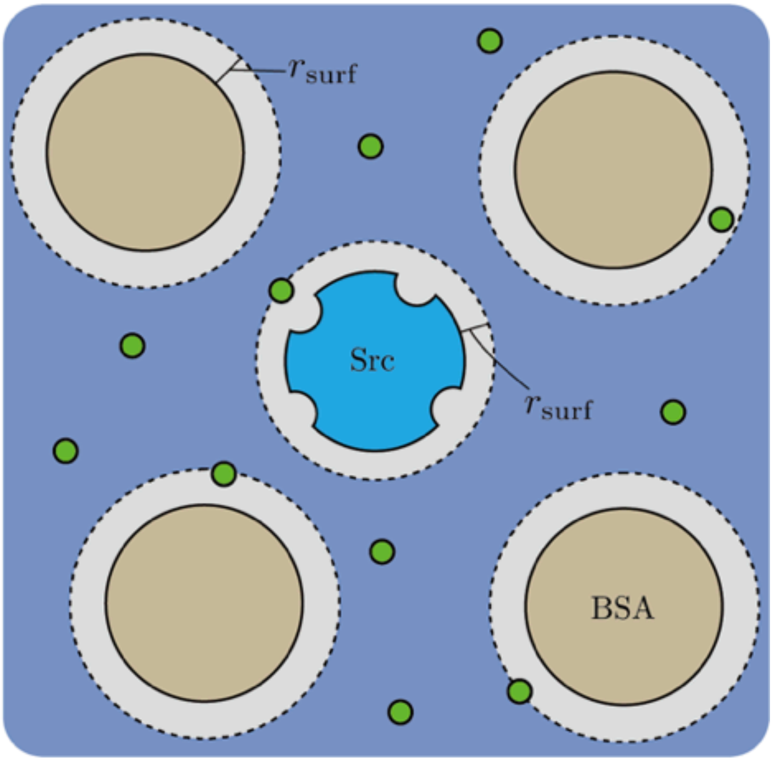

Supplementary Figure 8: The definition of the c-Src surface, BSA surface, and the bulk water regions. The c-Src kinase surface and BSA surface regions are defined if the minimum distance between L0 atom of PP1 and heavy atoms of the proteins is shorter than 5 Å. The region outside the surface regions is defined as the bulk water region.

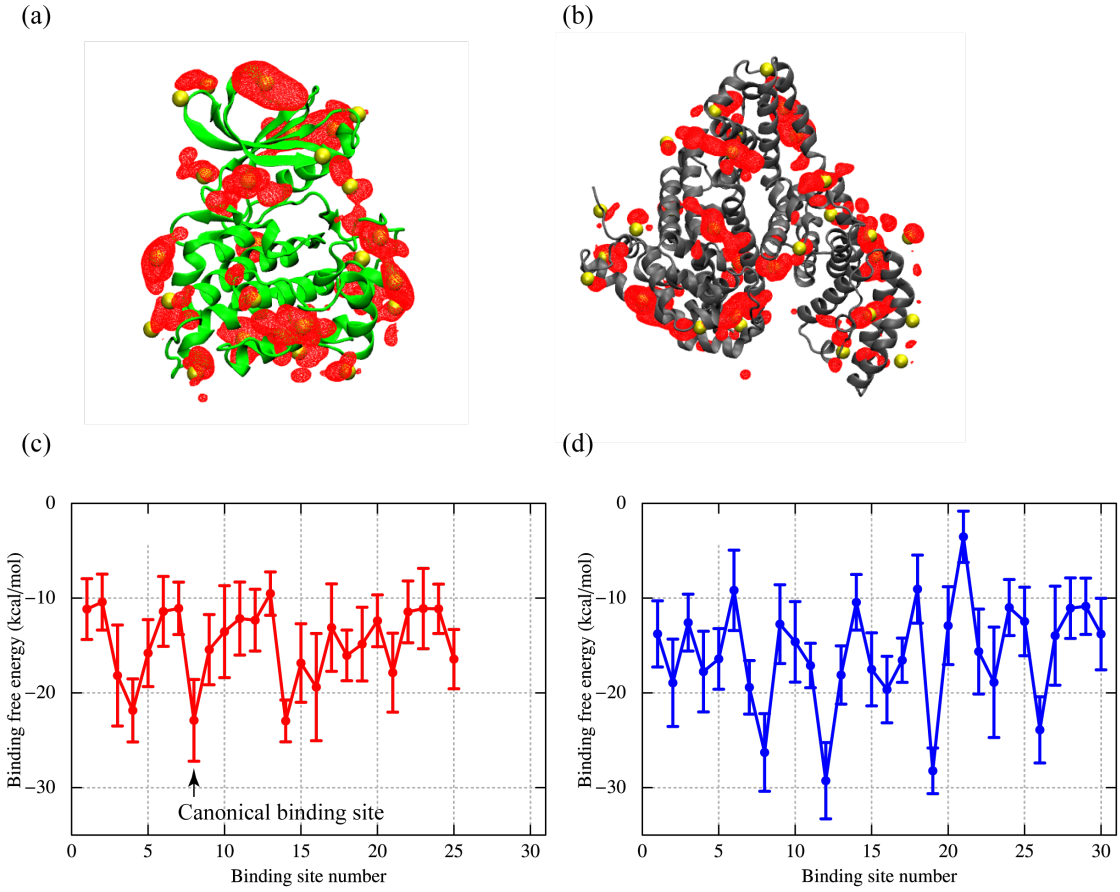

Supplementary Figure 9: Characterization of binding sites on c-Src kinase and BSA. The binding sites on c-Src kinase and BSA obtained from k-means++ clustering and the cluster centers are shown as yellow particles. The analysis is performed for (a) dilute and (b) Src2BSA systems. The binding free energies of (c) c-Src kinase-PP1 complex at the 25 binding sites and (d) BSA-PP1 complex at the 30 binding sites are calculated with the MM-GBSA analysis using AmberTools 18. Error bars represent the standard deviation from the mean. The number of snapshots for each binding site are described in Analysis Detail.

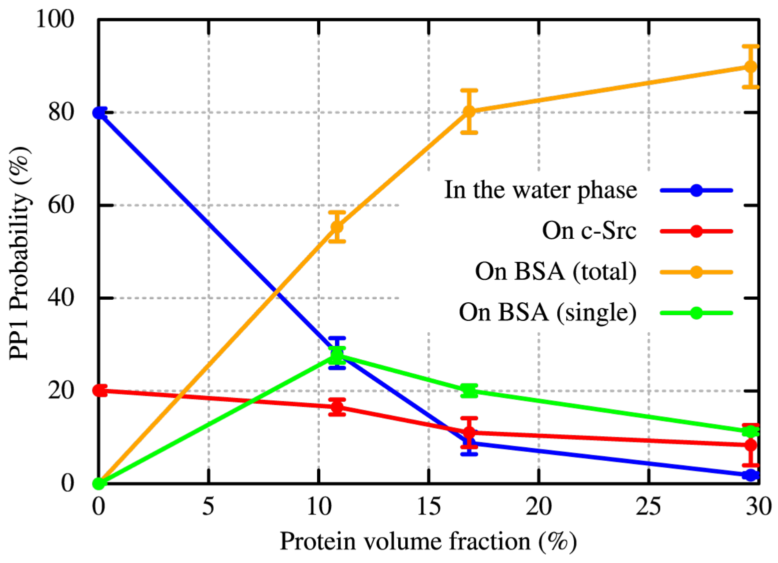

Supplementary Figure 10: The probability of finding PP1 in the bulk water region, on c-Src kinase, and on the BSAs as a function of the protein volume fraction. Error bars represent the standard error of the mean from seven trajectories for each system.

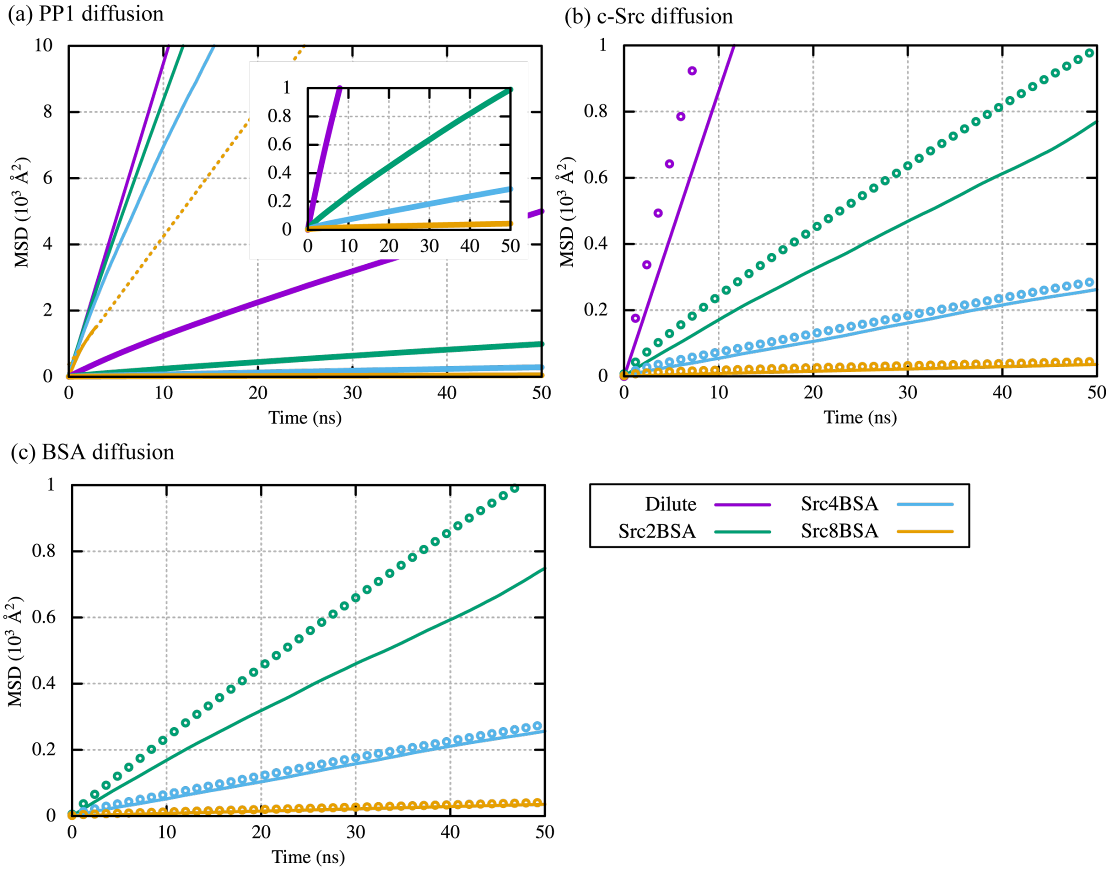

Supplementary Figure 11: Mean square displacement (MSD) of PP1, c-Src kinase, and BSA. (a) Spatially decomposed MSD of PP1 in the bulk water region (thin line), on the surface of c-Src kinase (thick line). In the case of the MSD in the bulk water region for Src8BSA system, the linear fitting of the MSD at $2 \text{ ns} \leq t \leq 3 \text{ ns}$ is shown as a dotted line. (b) MSD of c-Src kinase (solid line) together with that of PP1 on the c-Src kinase surface (circle). (c) MSD of BSA (solid line) together with that of PP1 on the BSA surface (circle).

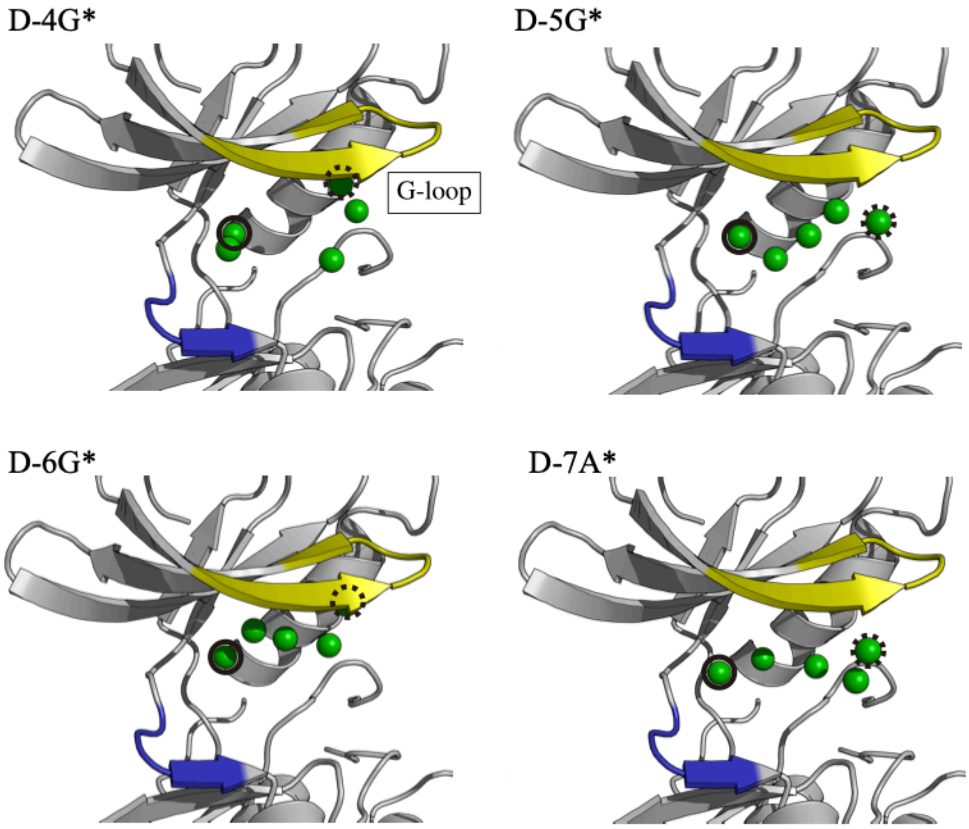

Supplementary Figure 12: Center-of-mass (CoM) binding trajectories in the dilute system. The position of CoM of PP1 along the binding trajectory is represented as green particle. Dashed and solid circles correspond to the starting and end (bound pose) points of each binding event, respectively.

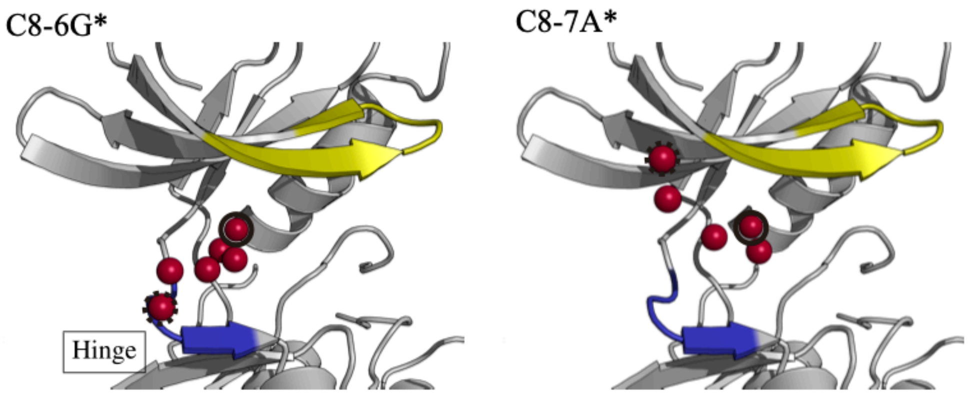

Supplementary Figure 13: Center-of-mass (CoM) binding trajectories in Src8BSA. The position of CoM of PP1 along the binding trajectory is represented as red particle. Dashed and solid circles correspond to the starting and end (bound pose) points of each binding event, respectively.

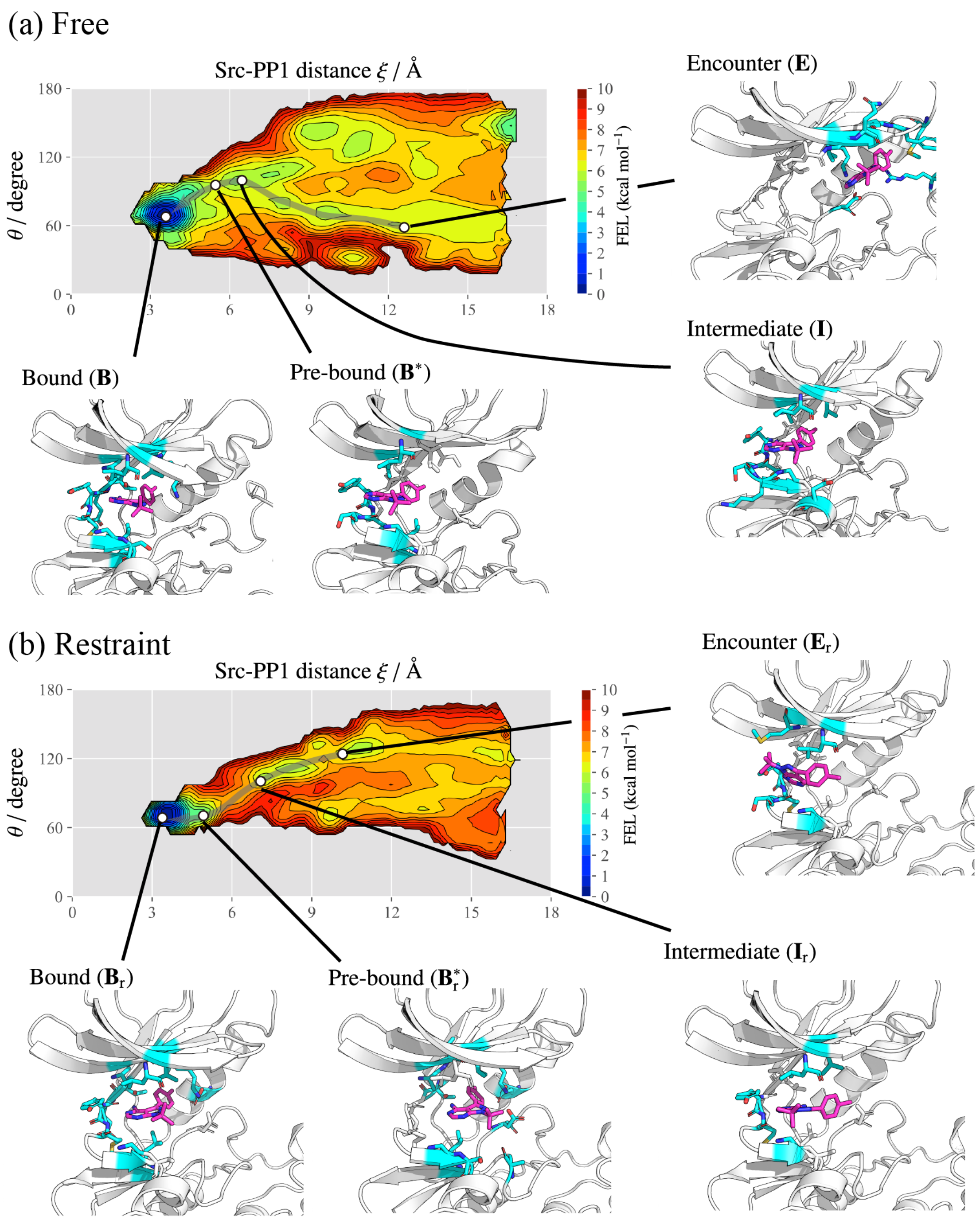

Supplementary Figure 14: Binding pathways predicted from gREST/REUS simulations under the dilute condition. (a) FEL without the positional restraints to c-Src kinase. (b) FEL with the positional restraints imposed on the residues outside the canonical binding site.

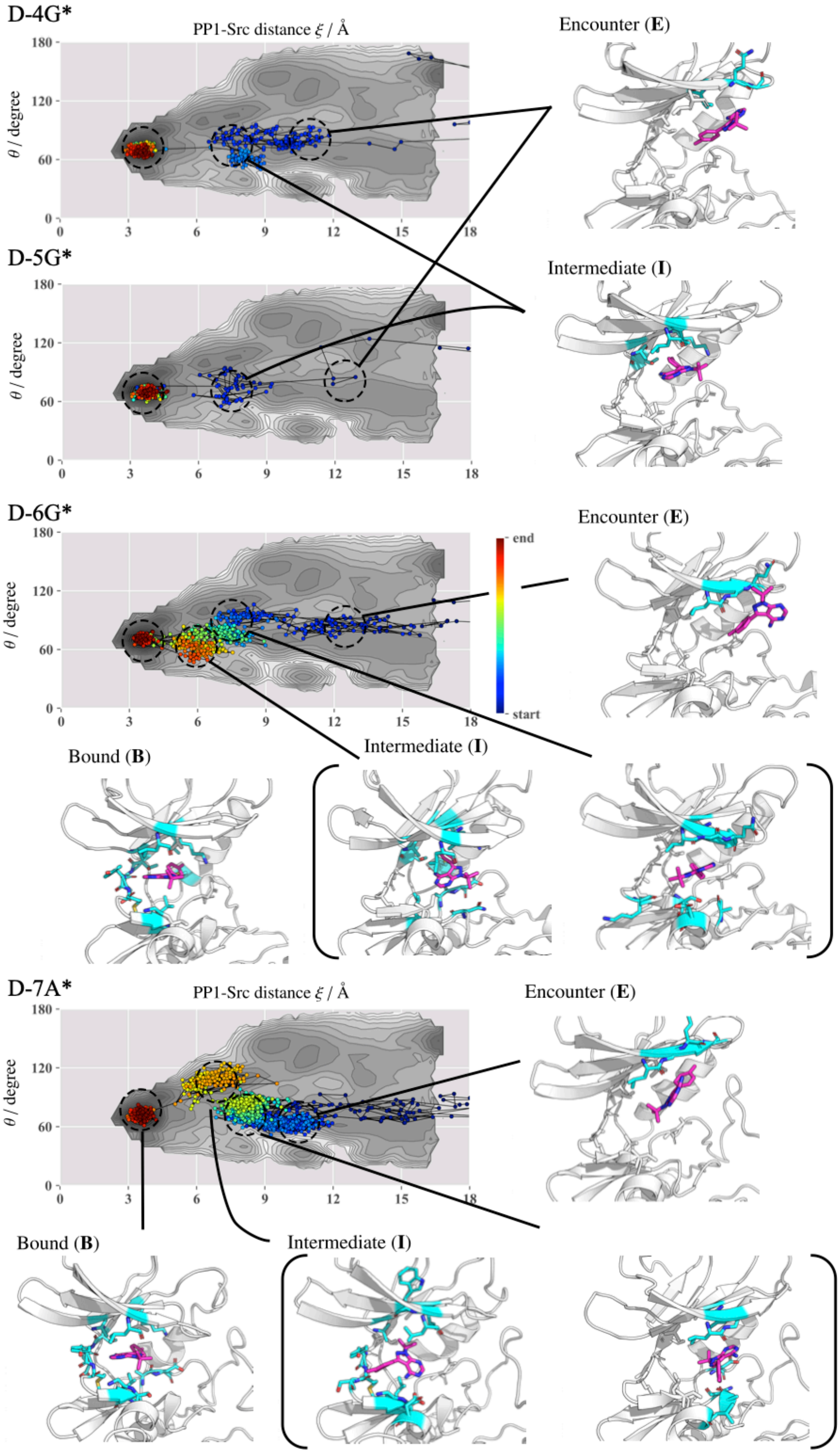

Supplementary Figure 15: Binding trajectories obtained from the present MD simulations in the dilute solution. Each of bound, intermediate, encounter state were defined in analogy with previous characterizations in gREST/REUS simulations[3].

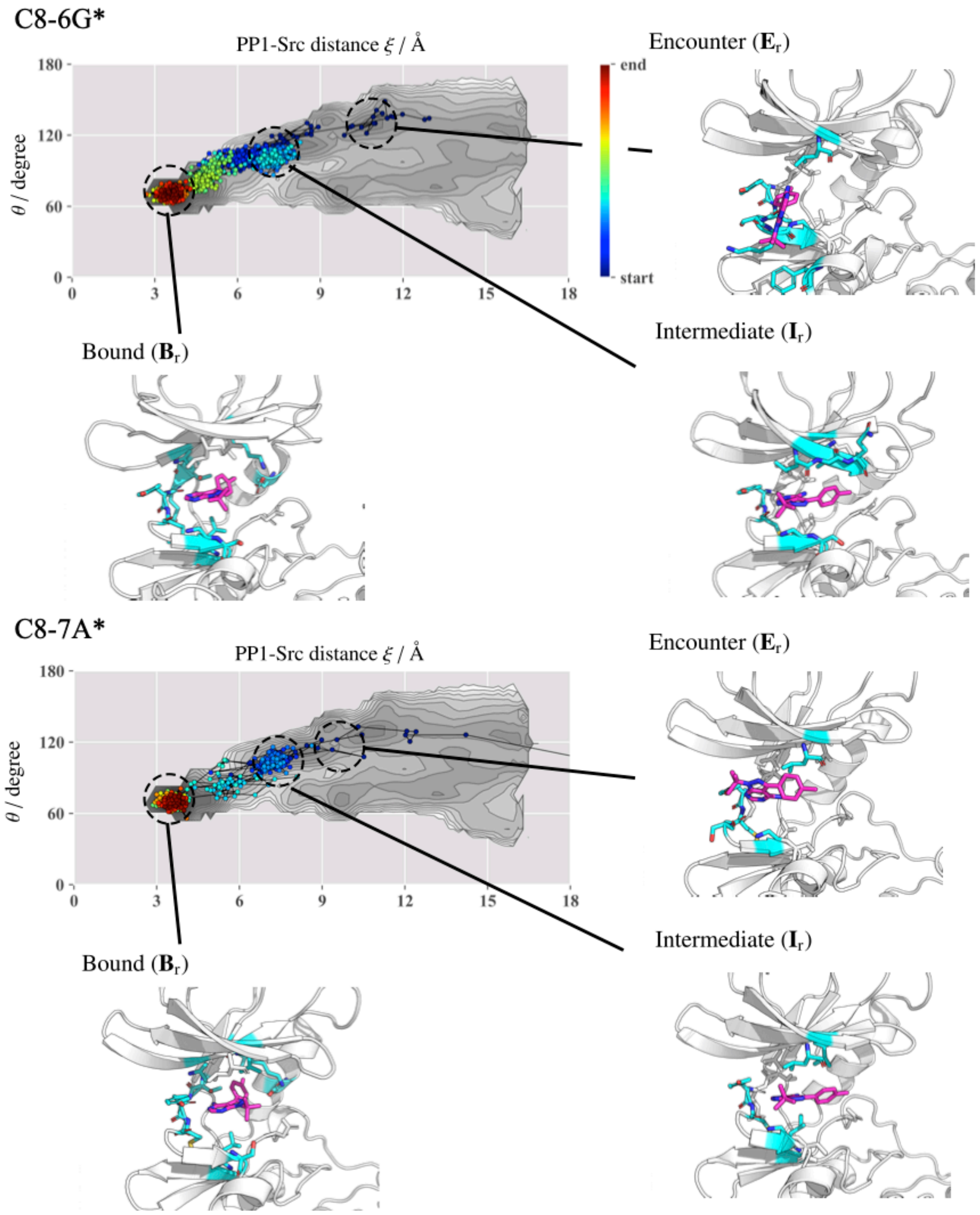

Supplementary Figure 16: Binding trajectories obtained from the present MD simulations in the crowded solution (Src8BSA). Each of bound, intermediate, encounter state were defined in analogy with previous characterizations in gREST/REUS simulations[3].

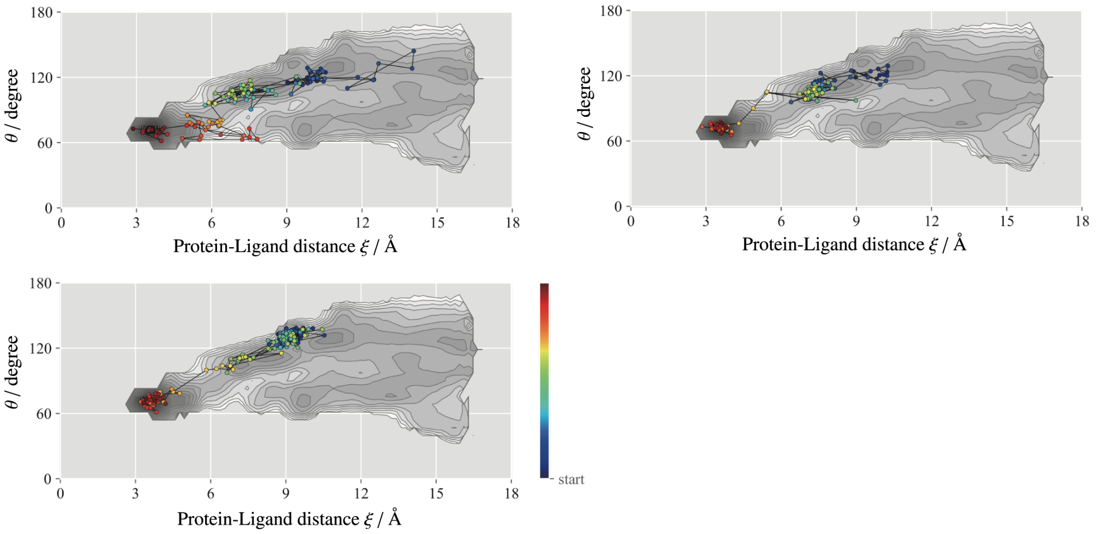

Supplementary Figure 17: Three binding trajectories starting from the $\mathbf{E_r}$ state in Src8BSA.

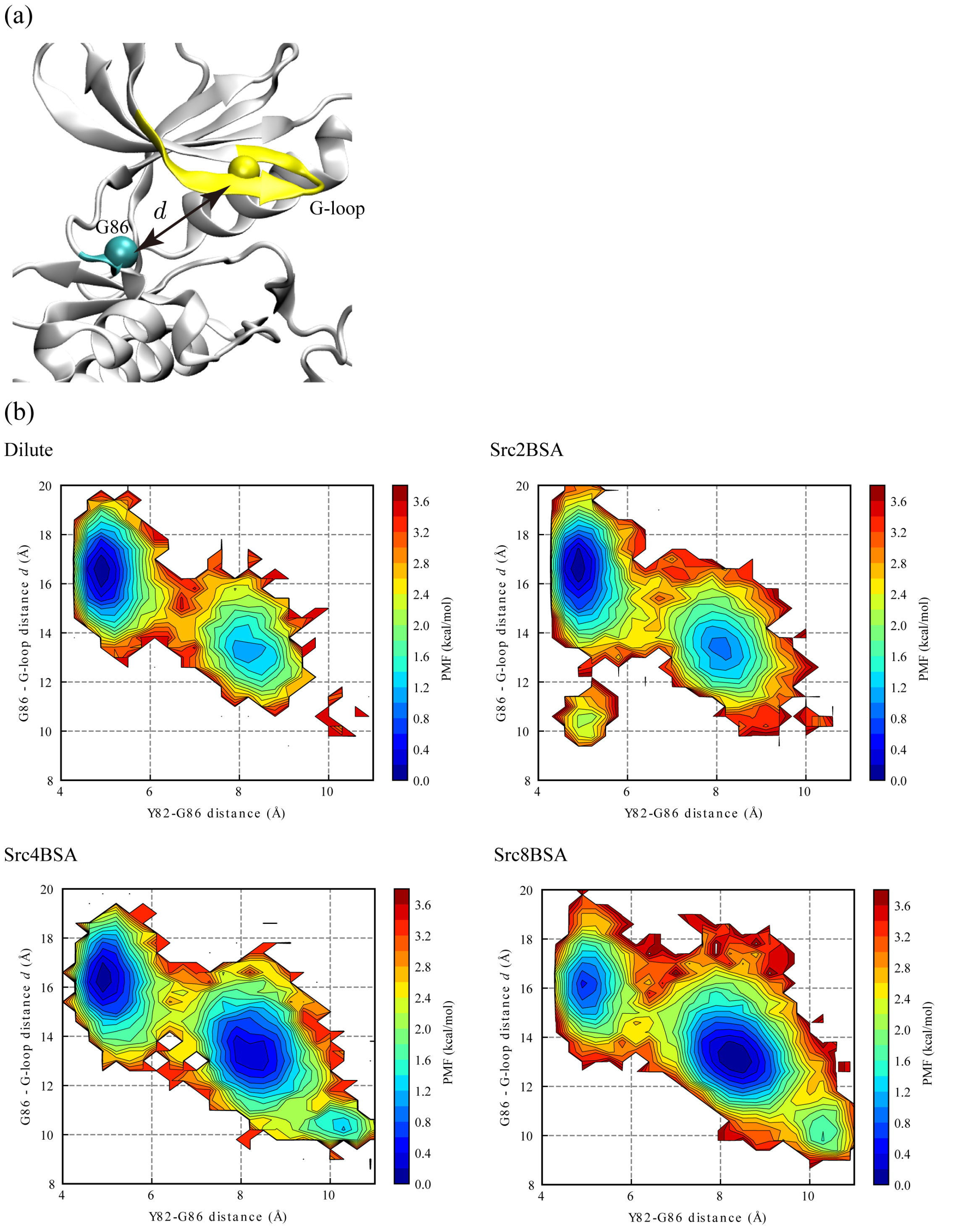

Supplementary Figure 18: Two-dimensional (2d) potentials of mean forces (PMFs) along with the distances between Tyr82 and Gly86 and between Gly86 and Center-of-mass (CoM) of G-loop. (a) The definition of distance between Gly86 and CoM of G-loop, which is defined using the Cα atoms of residues 14-22. (b) The 2d-PMFs for dilute, Src2BSA, Src4BSA, and Src8BSA in the clockwise direction.

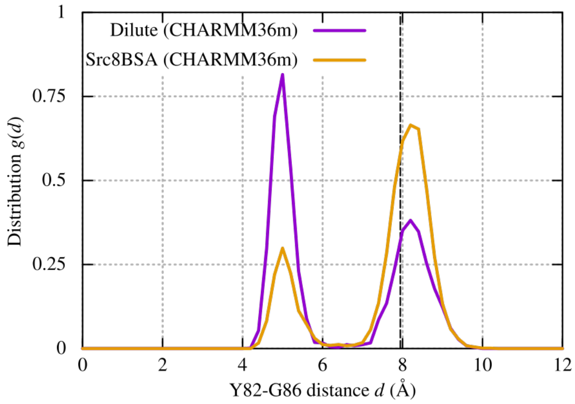

Supplementary Figure 19: Distribution for Tyr82-Gly86 distance, $g(d)$, obtained from the simulations in dilute and Src8BSA conditions using the CHARMM36m force field.

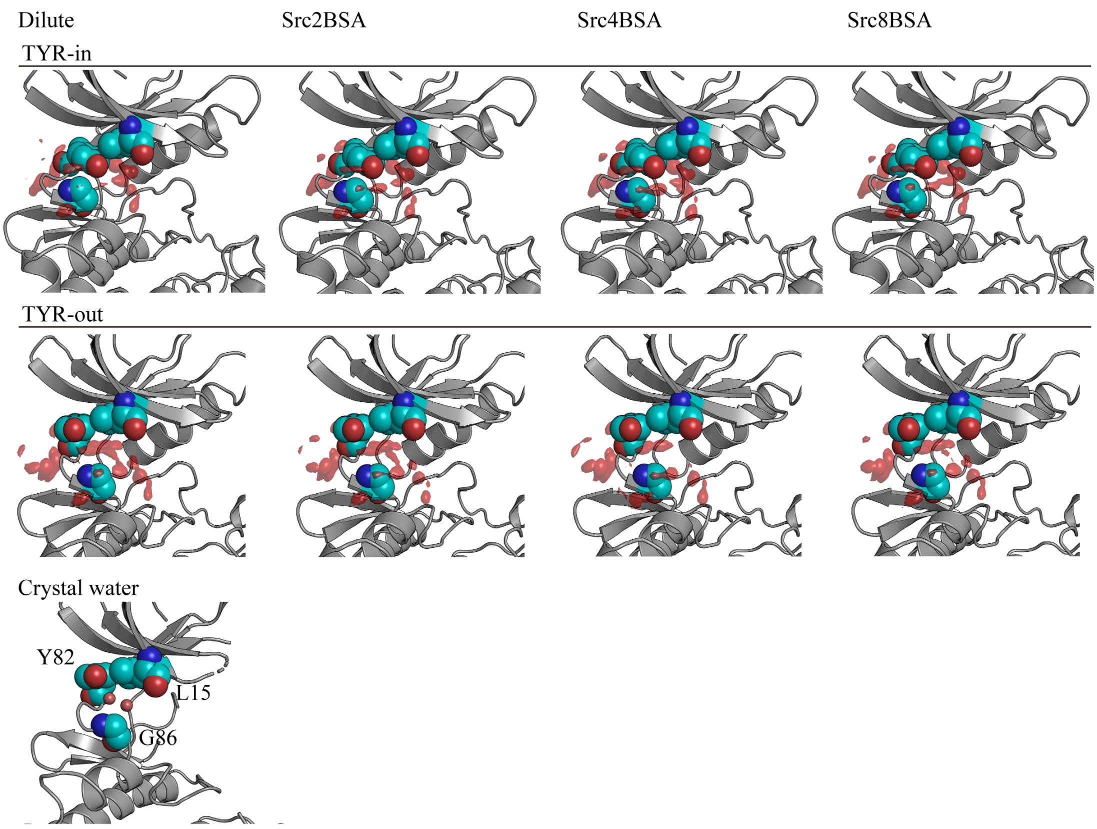

Supplementary Figure 20: Spatial distribution functions of water near the canonical binding site of c-Src kinase. The distributions were computed separately for TYR-in and TYR-out conformations in the dilute, Src2BSA, Src4BSA, and Src8BSA simulations. A water position between Leu15, Tyr82, and Gly86 in the crystal structure of c-Src kinase in the apo form (PDB ID: 1YOJ).[22]

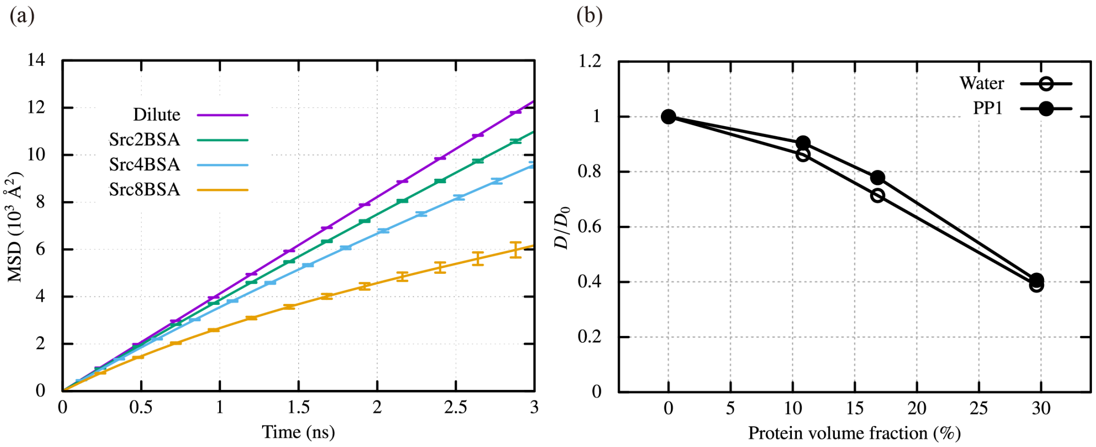

Supplementary Figure 21: (a) MSD of water molecules in the bulk water region. (b) The diffusion coefficient D of water molecules and PP1 in the bulk water region as a function of the protein volume fraction. The change is normalized with the diffusion coefficients in the dilute solution. The diffusion coefficients are obtained from the linear fitting of the MSDs at $2 \text{ ns} \leq t \leq 3 \text{ ns}$. In the calculation, 1000 water molecules were used in each simulation.

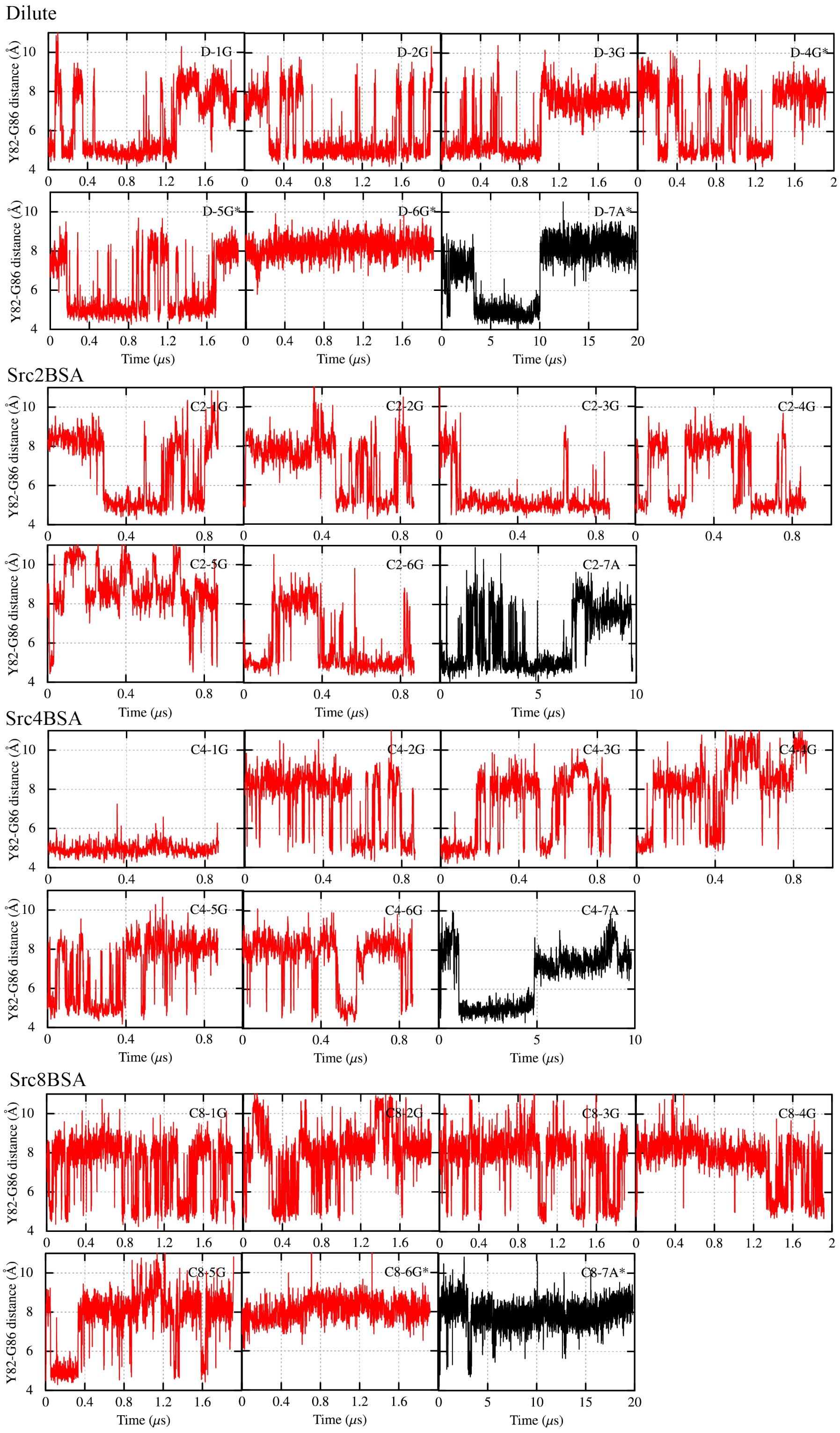

Supplementary Figure 22: Time series of the distance between Tyr82 and Gly86, which characterizes TYR-in and TYR-out conformations for dilute, Src2BSA, Src4BSA, and Src8BSA. The red lines were obtained using GENESIS, while the black ones using Anton2 in Pittsburg supercomputer center.

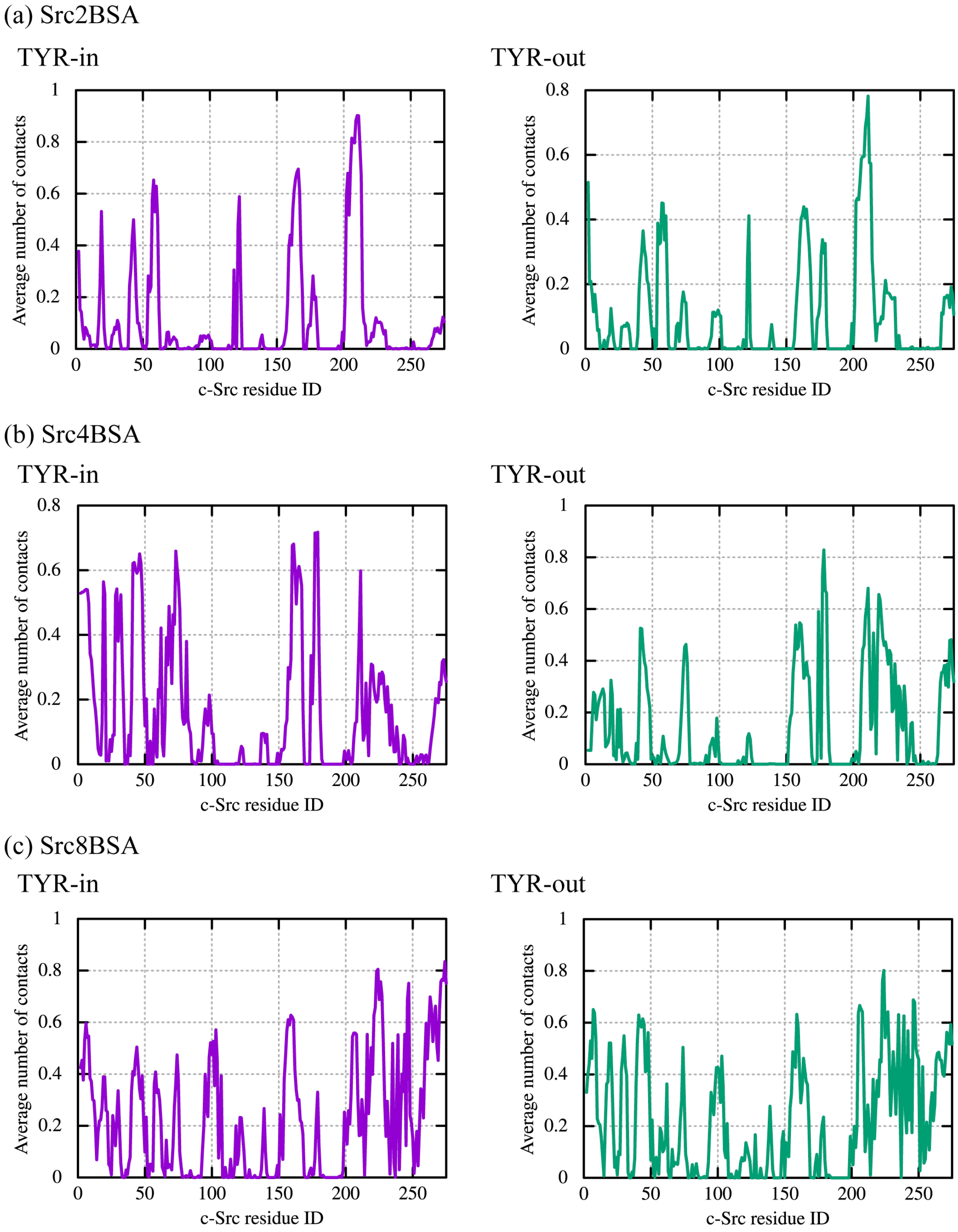

Supplementary Figure 23: Average number of the Cα atom contacts between BSA and c-Src kinase. A contact is defined if the minimum Cα atom distance between BSA and c-Src kinase is shorter than 10 Å.

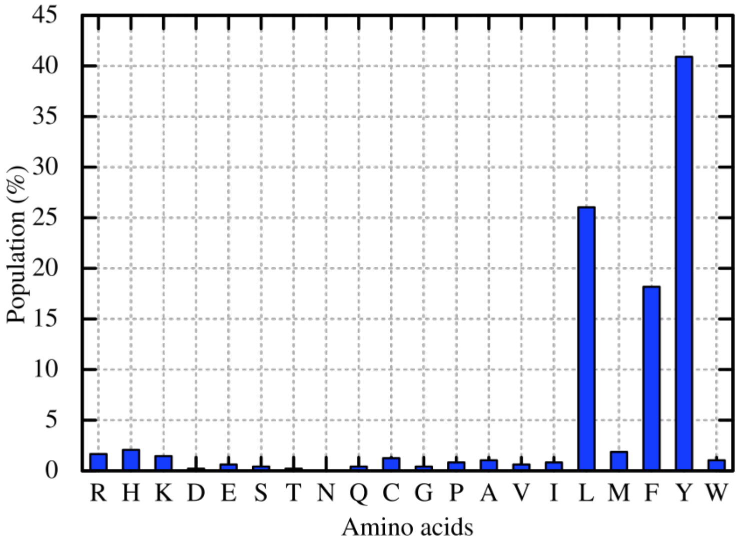

Supplementary Figure 24: Population of amino acids at the residue 82 among the variety of kinases. Sequence data analyzed here is taken from the reference 17.

Supplementary References

1. Cowan-Jacob, S. W. *et al.* The crystal structure of a c-Src complex in an active conformation suggests possible steps in c-Src activation. *Structure* **13**, 861–871 (2005).
2. Shan, Y. *et al.* How does a drug molecule find its target binding site? *J. Am. Chem. Soc.* **133**, 9181–9183 (2011).
3. Re, S., Oshima, H., Kasahara, K., Kamiya, M. & Sugita, Y. Encounter complexes and hidden poses of kinase-inhibitor binding on the free-energy landscape. *Proc. Natl. Acad. Sci.* **116**, 18404–18409 (2019).
4. Bujacz, A. Structures of bovine, equine and leporine serum albumin. *Acta Crystallogr. Sect. D - Biol. Crystallogr.* **68**, 1278–1289 (2012).
5. Lindorff-Larsen, K. *et al.* Improved side-chain torsion potentials for the Amber ff99SB protein force field. *Proteins Struct. Funct. Bioinforma.* **78**, 1950–1958 (2010).
6. Hornak, V. *et al.* Comparison of multiple Amber force fields and development of improved protein backbone parameters. *Proteins Struct. Funct. Bioinforma.* **65**, 712–725 (2006).
7. Jorgensen, W. L., Chandrasekhar, J., Madura, J. D., Impey, R. W. & Klein, M. L. Comparison of simple potential functions for simulating liquid water. *J. Chem. Phys.* **79**, 926–935 (1983).
8. Wang, J., Wolf, R. M., Caldwell, J. W., Kollman, P. A. & Case, D. A. Development and testing of a general amber force field. *J. Comput. Chem.* **25**, 1157–1174 (2004).
9. Ryckaert, J.-P., Ciccotti, G. & Berendsen, H. J. C. Numerical integration of the cartesian equations of motion of a system with constraints: molecular dynamics of n-alkanes. *J. Comput. Phys.* **23**, 327–341 (1977).
10. Miyamoto, S. & Kollman, P. A. Settle: An analytical version of the SHAKE and RATTLE algorithm for rigid water models. *J. Comput. Chem.* **13**, 952–962 (1992).
11. Jung, J. *et al.* GENESIS: a hybrid-parallel and multi-scale molecular dynamics simulator with enhanced sampling algorithms for biomolecular and cellular simulations. *Wiley Interdiscip. Rev. Comput. Mol. Sci.* **5**, 310–323 (2015).
12. Kobayashi, C. *et al.* GENESIS 1.1: A hybrid-parallel molecular dynamics simulator with enhanced sampling algorithms on multiple computational platforms. *J. Comput. Chem.* **38**, 2193–2206 (2017).
13. Shaw, D. E. *et al.* Anton 2: raising the bar for performance and programmability in a special-purpose molecular dynamics supercomputer. in *Proceedings of the international*

conference for high performance computing, networking, storage and analysis 41–53 (2014).

14. Bussi, G., Donadio, D. & Parrinello, M. Canonical sampling through velocity rescaling. *J. Chem. Phys.* **126**, 14101 (2007).
15. Huang, J. *et al.* CHARMM36m: an improved force field for folded and intrinsically disordered proteins. *Nat. Methods* **14**, 71–73 (2016).
16. Schindler, T. *et al.* Crystal structure of Hck in complex with a Src family--selective tyrosine kinase inhibitor. *Mol. Cell* **3**, 639–648 (1999).
17. Möbitz, H. The ABC of protein kinase conformations. *Biochim. Biophys. Acta (BBA)-Proteins Proteomics* **1854**, 1555–1566 (2015).
18. Humphrey, W., Dalke, A., Schulten, K. & others. VMD: visual molecular dynamics. *J. Mol. Graph.* **14**, 33–38 (1996).
19. Impey, R. W., Madden, P. A. & McDonald, I. R. Hydration and mobility of ions in solution. *J. Chem. Phys.* **87**, 5071–5083 (1983).
20. Case, D. *et al.* AMBER 18; 2018. *Univ. California, San Fr.*
21. Sekula, B., Zielinski, K. & Bujacz, A. Crystallographic studies of the complexes of bovine and equine serum albumin with 3, 5-diiodosalicylic acid. *Int. J. Biol. Macromol.* **60**, 316–324 (2013).
22. Breitenlechner, C. B. *et al.* Crystal structures of active SRC kinase domain complexes. *J. Mol. Biol.* **353**, 222–231 (2005).
