# Supplementary material for: Reduced efficacy of a Src kinase inhibitor in crowded protein solution
Source: Nat Commun. 2021 Jul 2;12:4099. doi: 10.1038/s41467-021-24349-5 (PMC8253829; doi:10.1038/s41467-021-24349-5)
Supplement: Supplementary file 5 — Description of additional supplementary files [file 41467_2021_24349_MOESM5_ESM.docx]

**Supplementary Movie 1:** A canonical binding event observed in the dilute solution (D-7A*)

**Supplementary Movie 2:** A canonical binding event observed in Src8BSA solution (C8-7A*)
